# Phosphate/Zinc Interaction Analysis in Two Lettuce Varieties Reveals Contrasting Effects on Biomass, Photosynthesis, and Dynamics of Pi Transport

**DOI:** 10.1155/2014/548254

**Published:** 2014-06-15

**Authors:** Nadia Bouain, Mushtak Kisko, Aida Rouached, Myriam Dauzat, Benoit Lacombe, Nibras Belgaroui, Tahar Ghnaya, Jean-Claude Davidian, Pierre Berthomieu, Chedly Abdelly, Hatem Rouached

**Affiliations:** ^1^Biochimie et Physiologie Moléculaire des Plantes, Institut National de la Recherche Agronomique, Centre National de la Recherche Scientifique, Université Montpellier 2, Montpellier SupAgro. Bat 7, 2 Place Viala, 34060 Montpellier Cedex 2, France; ^2^Laboratoire des Plantes Extrêmophiles, Centre de Biotechnologie de Borj Cédria, BP 901, 2050 Hammam-Lif, Tunisia; ^3^Laboratoire d'Ecophysiologie des Plantes Sous Stress Environnementaux, UMR 759, INRA/SUPAGRO, 34060 Montpellier Cedex 1, France; ^4^Laboratoire de Protection et Amélioration des Plantes, Centre de Biotechnologie de Sfax, BP 1177, 3018 Sfax, Tunisia

## Abstract

Inorganic phosphate (Pi) and Zinc (Zn) are essential nutrients for normal plant growth. Interaction between these elements has been observed in many crop plants. Despite its agronomic importance, the biological significance and genetic basis of this interaction remain largely unknown. Here we examined the Pi/Zn interaction in two lettuce (*Lactuca sativa*) varieties, namely, “Paris Island Cos” and “Kordaat.” The effects of variation in Pi and Zn supply were assessed on biomass and photosynthesis for each variety. Paris Island Cos displayed better growth and photosynthesis compared to Kordaat under all the conditions tested. Correlation analysis was performed to determine the interconnectivity between Pi and Zn intracellular contents in both varieties. Paris Island Cos showed a strong negative correlation between the accumulation levels of Pi and Zn in shoots and roots. However, no relation was observed for Kordaat. The increase of Zn concentration in the medium causes a decrease in dynamics of Pi transport in Paris Island Cos, but not in Kordaat plants. Taken together, results revealed a contrasting behavior between the two lettuce varieties in terms of the coregulation of Pi and Zn homeostasis and provided evidence in favor of a genetic basis for the interconnection of these two elements.

## 1. Introduction

Zinc (Zn) and phosphorous (P) are important micro- and macronutrients required for optimal plants growth [1–4]. Plants absorb these elements from the soil solution using root system. Often the concentration of these elements in agriculture soil is very low, thus causing Zn and Pi deficiency in plants which negatively affects plants metabolism and photosynthesis [5]. Worldwide agriculture has become dependent on external sources of Zn and Pi fertilizers in order to address the issue of sustainable food resources for the growing world population. Nevertheless, this strategy has adverse economic and ecological impacts, particularly for Pi. It is predicted that high-grade and easily-extractable Pi from rocks will be exhausted [6]. Therefore, substantial efforts have been made to improve Zn and Pi nutrition in plants based on our current understanding on how plants respond to the deficiency of each individual element. However, in practice, application of such knowledge is hindered by complex cross-talks, which are emerging in the face of evidences overwhelmingly showing that Zn and Pi nutrition are interrelated, which likely to sustain plants growth and development. Lines of evidences support the fact that Pi-Zn interaction occurs within the plant [7–12]. Such interconnections have consequences on comprehending the regulation of Zn and Pi homeostasis and can account for shortcomings of current agronomic models that are typically focused to improve the assimilation of individual elements.

Zn availability or its absence in the medium can either increase or decrease the accumulation of Pi in plants, respectively [10, 12, 13]. The positive effects of Zn deficiency on the Pi uptake by roots and its overaccumulation in leaves have been observed in numerous plant species such as tomato [14], okra [15] and cotton [7], and barley [9]. Lately such effect was reported in Arabidopsis [12]. The specificity of the Zn-Pi relationship has been further demonstrated by the fact that in barley only Zn deficiency could induce Pi uptake and not nitrogen, sulfur, nor manganese deficiency [9]. Similarly, cotton or tomato plants do not show an overaccumulation of Pi under iron or copper deficiency [7, 16]. It seems that plants lose the capacity to regulate Pi homeostasis under Zn deficiency and can overaccumulate Pi in shoots under high Pi concentrations leading to phytotoxic symptoms [7]. Excessive Zn application has been shown to decrease Pi concentration in plants [17, 18]. Nevertheless, the underlying mechanisms for the Pi-Zn homeostasis interaction* in planta* remain to be deciphered, which is of primary importance to improve the Pi and Zn nutrition in vegetable crops using agronomical/biotechnological programs together with an appropriate fertilizer management schemes.

Among cultivated plant species, lettuce* Lactuca sativa* (family* Asteraceae*) is a major vegetable in western countries [19]. Lettuce is the second most consumed fresh vegetable in the USA at 28.0 pounds per capita in 2008, behind potato at 36.7 pounds [19]. Lettuce has limited roots and rapid top growth requires high levels of P supply for maintaining proper growth. As aforementioned, the excessive use of Pi fertilizers contributes in lowering Zn concentrations in soil [20] and more adversely can favor the uptake of other heavy metals [21–23]. Thus improving the Pi use efficiency, while maintaining an appropriate level of Zn in lettuce, is of primary importance for sustainable agriculture. Lettuce varieties with contrasting features for essential nutrients accumulation may constitute a good plant material to study the Pi and Zn nutrition.

Two lettuce varieties, namely, Paris Island Cos and Kordaat, exhibiting contrasting features for the characters of heavy metal accumulation [23] were considered to investigate Zn and Pi interaction. The effects of the Pi and/or Zn treatments on the growth capacity and accumulation of these ions in the shoots and roots were determined. Photosynthesis and stomatal conductance was assessed under each stress condition. Zn deficiency influence on Pi uptake and translocation in the lettuce varieties was studied using ^33^P isotope. Results revealed differential regulation of Zn-Pi homeostasis interaction in the two lettuce varieties.

## 2. Materials and Methods

### 2.1. Plant Material and Growth Conditions

Two varieties of* L. sativa *(lettuce) considered for this work were Paris Island Cos and Kordaat. Lettuce seeds were germinated on top of humidified paper (Whatman) with distilled water for 3 days and then with modified Hoagland nutrient (2.5 mM KNO_3_, 0.5 mM NaH_2_PO_4_, 2.5 mMCa(NO_3_)_2_, 0.5 mM MgSO_4_, 0.1 mM FeIIINaEDTA, 0.05 mM H_3_BO_3_, 0.05 mM MnSO_4_, 15 *μ*M ZnSO_4_, 3 *μ*M Na_2_MoO_4_, 2.5 *μ*M KI, 0.05 *μ*M CuSO_4_, and 0.044 *μ*M CoCl_2_) for 7 additional days. Seedlings were carefully transferred to 9-L tanks containing the same nutrient solution. 10 days later, plants were treated with different Zn (0, 15, 90, 360, 1440, and 2880 *μ*M) and Pi (0 and 500 *μ*M) concentrations for eight additional days. Plants were grown in a growth chamber under the following environmental conditions: light/dark cycle of 8 h/16 h with light intensity being 250 *μ*mol*·*m^−2^ *·*s^−1^, temperature of 20°C, and relative humidity of 65%. Nutritive solutions were renewed every 4 days during the whole experiment. Analyses were performed on separated shoots and roots of individual plants.

### 2.2. Zinc and Phosphate Contents Measurement

Zn concentration was determined using dried plant samples. The digestion and extraction were done using hydrogen peroxide and nitric acid as described in [23]. Concentrations of Zn in the extracts were determined by atomic absorption spectrophotometry (SpectrAA 220, Varian, Australia). Pi measurements were performed as described by [24]. Briefly, the extraction was performed on fresh shoots and roots samples by incubating in ultrapure water at 70°C for 30 min. Pi content was evaluated by colorimetry at 820 nm using the molybdate assay, according to the procedure of [25].

### 2.3. Phosphate Uptake and Transfer Measurements

Phosphate uptake and root-to-shoot transfer measurements were performed using whole lettuce plants grown hydroponically, after germination stage for ten days, and for additional ten days in different Zn concentrations (0, 15, 90, and 180 *μ*M) containing 500 *μ*M PO_4_^2−^. For root influx and root-to-shoot translocation, roots of whole plants were placed in Na_2_PO_4_ solution at pH 5.0 in the presence of 10 *μ*Ci/mL of the radiotracer ^33^P-Orthophosphoric Acid (PerkinElmer) for 5 min and 2:30 h, respectively. Lettuce plants were then washed in an ice-cold 5 mM Na_2_PO_4_ solution and then shoots and roots were harvested separately, dried, and the radioactivity was measured using scintillation counting [24]. Root-to-shoot Pi transport was expressed as the percentage of radioactivity located in the shoot over the total amount of radioactivity in the whole lettuce plant.

### 2.4. Measurements of Photosynthesis and Stomatal Conductance

Leaf net photosynthetic rate (A, *μ*mol fixed CO_2_ m^−2^ *·*s^−1^) and stomatal conductance (B, mol*·*m^−2^ *·*s^−1^) rates were measured on the third fully expanded leaves using a portable photosynthesis system with a light-emitting diode light source (LI-6400, LI-COR, Inc.; Lincoln, NE) according to the manufacturer's protocol. Experiments were performed under controlled conditions (20°C, 65% relative humidity, and controlled CO_2_ supply of 400 *μ*mol mol^−1^) with photon flux density 225 *μ*mol*·*m^−2^ s^−1^.

### 2.5. Statistical Analysis

Data are presented as means of at least three independent experiments and mean values were taken for statistical analyses using one-way ANOVA. Significant differences were further analyzed using Tukey's parametric or nonparametric tests to identify differences between treatments and/or varieties. The differences were considered significant if *P* ≤ 0.05.

## 3. Results and Discussion

### 3.1. Paris Island Cos and Kordaat Varieties Displayed Differential Biomass Production and Photosynthesis

**Figure 1:**
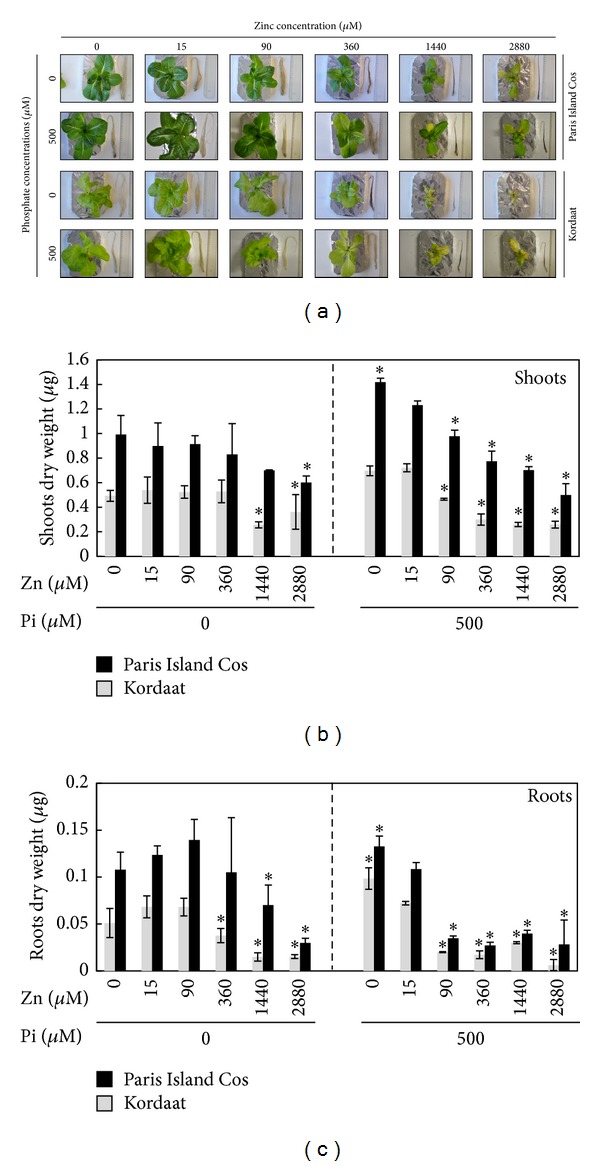
Zinc and phosphate treatment significantly alters Paris Island Cos and Kordaat growth capacity. 30-day-old Paris Island Cos and Kordaat lettuce varieties were grown and exposed to various zinc and phosphate concentrations in the culture medium (a). Shoot (b) and root (c) dry weight measured in different growth conditions for the Paris Island Cos and Kordaat lettuce varieties. Results are averages of three replicates ± SE. Asterisks indicate statistically significant differences compared to either 0 *μ*M of Pi, 15 *μ*M Zn (left parts of (b) and (c)) or 500 *μ*M of Pi, 15 *μ*M Zn (right parts of (b) and (c)) treatments of each lettuce variety (*P* ≤ 0.05).

**Figure 2:**
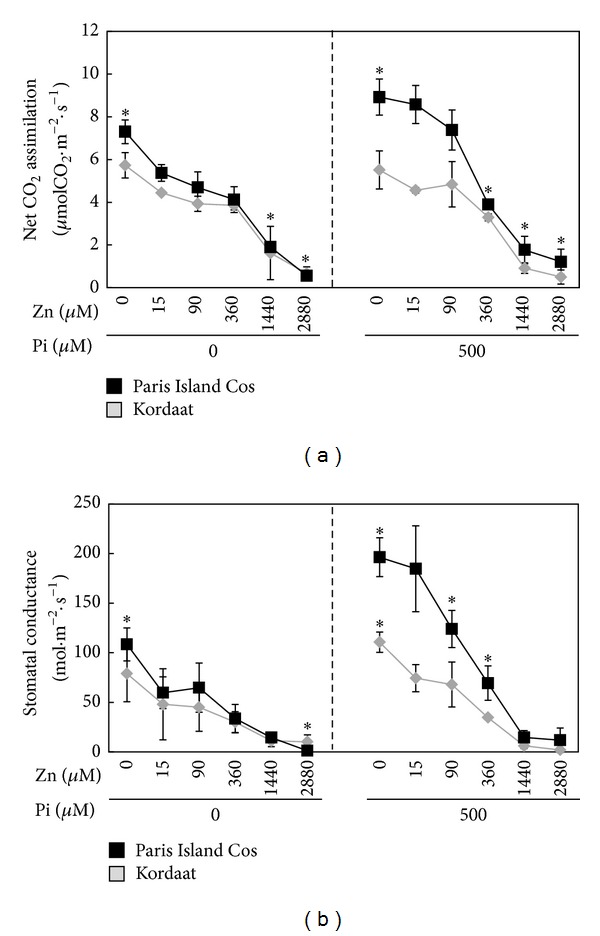
Varying zinc and phosphate concentrations in the culture medium alter the photosynthetic potential of the two lettuce varieties. Photosynthesis (a) and stomatal conductance (b) were measured for the Paris Island Cos and Kordaat lettuce varieties on third fully expanded leaves of each plant. Individual measurements were obtained from a pool of “*n*” plants (*n* ≥ 3). Error bars indicate SD.

The Zn availability can adversely alter plant growth in situations where it is present in either too low (deficiency) or too high (toxicity) concentration [16]. The plant biomass production is also altered in case of low Pi supply [2]. Such symptoms are often described without taking into account the bioavailability of one or the other element. Herein, we investigated the effects of varying external concentrations of Zn and/or Pi on the growth capacity of two lettuce varieties, namely, Paris Island Cos and Kordaat. Lettuce plants were grown hydroponically under control condition (500 *μ*M Pi and 15 *μ*M Zn) for one week then transferred on 12 different mediums resulting from the combination of two Pi (0 and 500 *μ*M) and six Zn (0, 15, 90, 360, 1440, and 2880 *μ*M) concentrations. The dry weight of 4-week-old plants were determined (Figure 1). Results show that the increase of Zn concentration in the medium leads to the reduction of shoot and root dry weight in both varieties (Figures 1(b) and 1(c)). The most visible Zn toxicity symptoms included decrease in leaf size and appearance of necrosis (Figure 1(a)). Under high Zn concentration (1,440 mM and 2,880 mM), no shoots growth pattern was observed for both varieties (Figure 1(a)). The presence or absence of Pi from the medium mitigates or aggravates the Zn toxicity (Figure 1(a)). Nevertheless, overall Paris Island Cos displayed a better growth capacity compared to Kordaat under all the conditions tested (Figure 1). A part of the explanation of the observed effects of Pi and Zn nutrition on the growth capacity of both lettuce varieties could be an alteration in photosynthesis. Indeed, both elements are known for their influences on this vital process. On one hand, Pi participates in plant photosynthesis in the form of ATP, which supplies energy for the CO_2_ fixation [26]. Zn role as an essential constituent of enzymes important to photosynthesis, such as the carbonic anhydrase, is well documented [27]. Zn excess affects the photochemical reactions of the photosystems [27]. To test this hypothesis, the net CO_2_ assimilation and stomatal conductance were assessed in Paris Island Cos and Kordaat grown on the aforementioned 12 growth conditions. Results revealed that in contrast to Kordaat plants, the photosynthesis was severely affected in Paris Island Cos in response to Pi deficiency and showed a strong reduction when grown under 0 *μ*M Pi and 15 *μ*M Zn compared to control condition (500 *μ*M Pi and 15 *μ*M Zn) (Figure 2). This result is in agreement with previous one in maize (*Z. mays*) or with bean (*P. vulgaris*) plants, in which plants grown in low Pi condition resulted in decline of the photosynthetic rate by 68% and 50% compared to the control plants, respectively [28, 29]. Results presented in Figure 2(a) shows that net CO_2_ assimilation decreased with increasing Zn concentration in the medium for both lettuce varieties. These observations suggest that the registered growth reduction for both lettuce varieties is the overall effect of Zn toxicity which may be related to the major effect of Zn toxicity on inhibition of photosynthesis. Interestingly, the presence of Pi contributes to the alleviating effect of Zn excess on this process. While both varieties behave similarly regardless of Zn concentration in the absence of Pi (Figures 2(a) and 2(b)), they exhibited contrasting behavior in presence of Pi. Paris Island Cos grown in presence of 500 *μ*M of Pi and 0, 15, or 90 *μ*M of Zn showed significantly higher CO_2_ assimilation and stomatal conductance than Kordaat. Although high Zn concentration strongly reduced the above parameters in both varieties, overall Paris Island Cos was more superior than Kordaat which can be credited to its improved growth performance and likely to a better Pi use efficiency (Figures 1(a), 1(b), and 1(c)).

### 3.2. Effect of Zn and Pi Supply on Their Endogenous Content in Shoots and Roots

**Figure 3:**
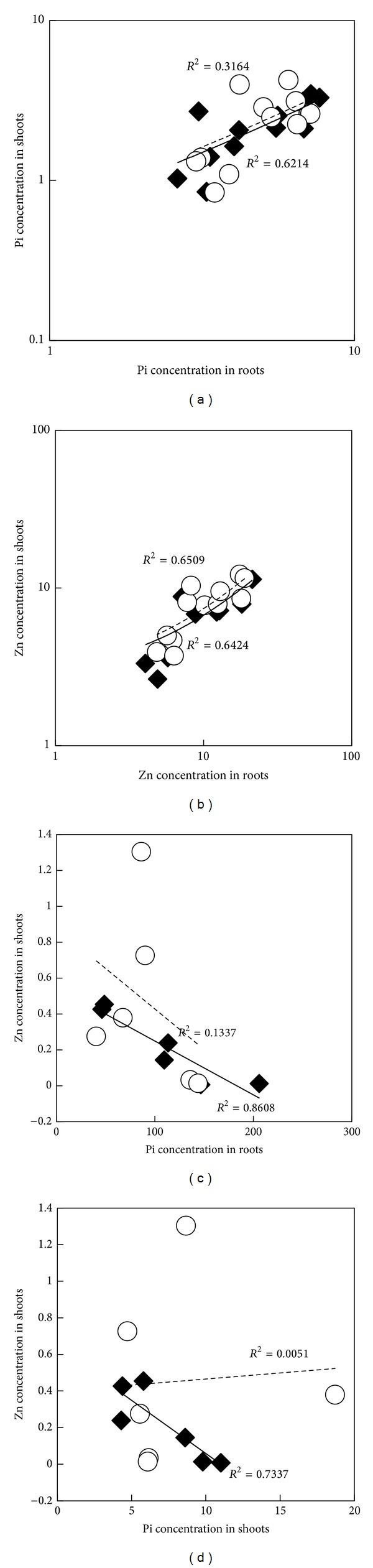
Correlation between zinc and inorganic phosphate in Paris Island Cos and Kordaat. Zinc and inorganic phosphate contents were determined in shoots and roots of the two lettuce varieties (◯, Kordaat; ◆ Paris Island Cos) grown in the presence of 500 *μ*M of Pi and changing Zn concentrations (0, 15, 90, 360, 1440, or 2880 *μ*M). Correlation between Pi content in shoots and Pi content in roots (a). Correlation between Zn content in shoots and roots (b) Correlation between Zn content in shoots and Pi content in roots (c). Correlation between Zn content in shoots and Pi content in shoots (d). Lines correspond to linear regression. For each regression, the square of Pearson's correlation coefficient (*R* ^2^) is reported.

As already mentioned, earlier studies have reported that Zn deficiency leads to the overaccumulation of Pi in the shoot, and inversely [12, 13]. In different plant species, a negative correlation between tissue Pi and Zn treatments has been observed. However, the nature of this correlation in conditions where Zn and/or Pi treatments vary from the depletion to the excess in the medium is poorly documented. We have determined the accumulation level of intracellular Zn and Pi contents in the two lettuce varieties. The observed typical Zn toxicity symptoms described above (Figure 1(a)) correspond to the highest shoot Zn accumulation in both of the varieties. The Zn overaccumulation can be ascribed to an impaired control mechanism of Zn uptake and release from root cells to xylem due to the altered morphology of roots under Zn excess in the medium [30, 31]. Such dysfunctional roots can also explain the high Pi accumulation in roots exposed to high Zn concentration (Table 1). Under control conditions, (15 *μ*M Zn; 500 *μ*M Pi) the Paris Island Cos and Kordaat accumulated 9.79 ± 0.15 *μ*mol*·*g^−1^ FW and 6.11 ± 0.9 *μ*mol*·*g^−1^ FW of Pi, respectively. Both varieties accumulate similar Zn concentrations in shoots (*≈*0.01 *μ*g*·*g^−1^ DW). Interestingly, Zn treatment was found to significantly affect the shoots Pi content in Paris Island Cos which got decreased as the Zn concentration increased in the medium (Table 1). Pi content was at maximum under Zn deficiency (11.02 ± 2.18 *μ*mol*·*g^−1^ FW) and dropped significantly (4.31 ± 0.17 *μ*mol*·*g^−1^ FW) in presence of 1,440 mM of Zn (Table 1). This result is in agreement with previous studies that have shown that Zn deficient plants can overaccumulate Pi, such as cotton [7] and barley [9]. Results revealed that variation in Zn supply affected differentially the accumulation of Pi in the shoot and roots of the two lettuce varieties. A correlation analysis was performed to determine the interconnectivity of Zn and Pi in the plants (Figure 3). Interestingly, a strong correlation was found between the Zn concentration in the shoot and the Pi concentration in either the root or the shoot of Paris Island Cos (Figures 3(c) and 3(d)), but not in Kordaat variety. Our data thus supported the role of shoot Zn content on the regulation of Pi content in Paris Island Cos, but not for the Kordaat variety. The contrasting behavior between Paris Island Cos and Kordaat may be explained by a difference in regulating Pi uptake and translocation in response to Zn availability. These results revealed that Pi-Zn homeostasis interaction may vary even within the same plant species, which pave the way for a genetic study for cloning quantitative trait loci (QTL)/gene(s) governing these traits. This approach has been successfully used to identify QTL for Pi and Zn in wheat, which appeared to be colocalized [32].

### 3.3. Differential Effect of Zn Supply on Pi Transport Dynamic in Paris Island Cos and Kordaat

**Figure 4:**
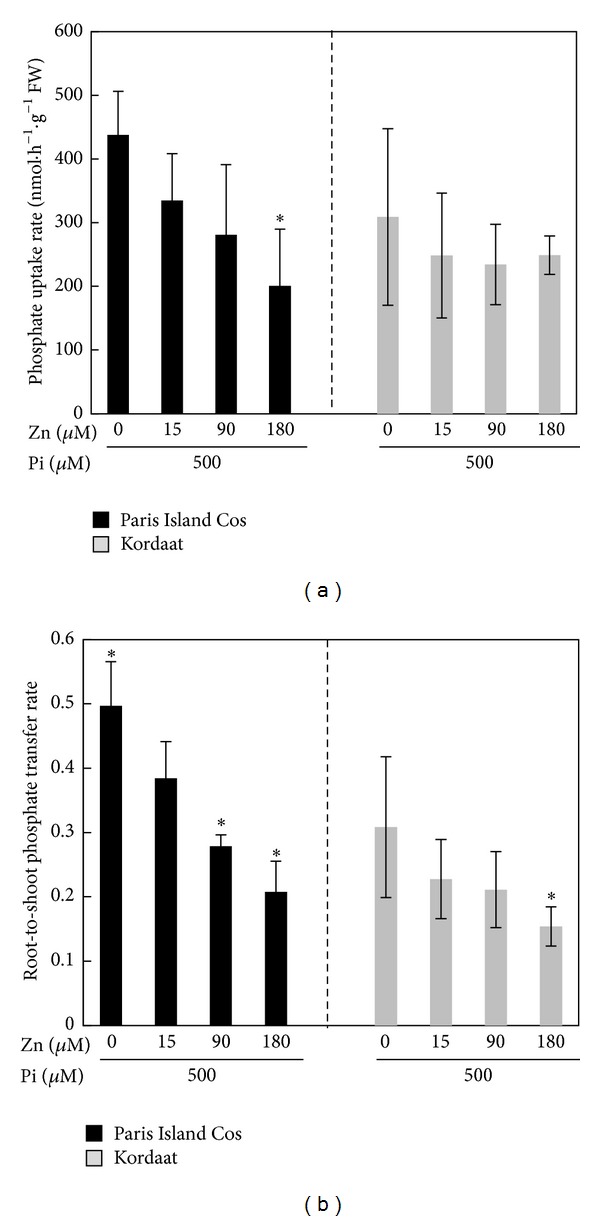
Zinc deficiency affects phosphate transport dynamics. Paris Island Cos and Kordaat lettuce varieties were grown hydroponically in media with various concentrations of zinc and inorganic phosphate (Pi). Plant uptake is defined as *μ*mol of Pi acquired by the whole plant per g of root fresh weight per hour. Pi root-to-shoot transfer is defined as the ratio of radioactive Pi in the shoot over the total radioactive Pi in the plant. Individual measurements were obtained from the analysis of shoots or roots collected from a pool of “*n*” plants (*n* ≥ 3). Error bars indicate SD.

**Figure 5:**
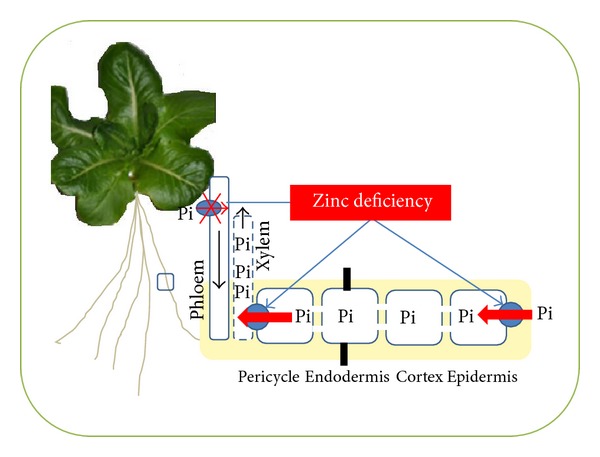
Schematic representation of the regulation of Pi transport within lettuce plant; case of Paris Island Cos. Pi is acquired into root by PHT1s. Transport into the xylem, likely through the Pi exporter PHO1s. The Zn deficiency leads to the increase of Pi uptake and is loading into root xylem. Zn deficiency might lead also to the inhibition of Pi shoot-to-root transfer via unknown protein.

It has been proposed that Zn deficiency may depress root Pi uptake and may also be involved in a high rate of Pi transfer to the shoot, leading to its overaccumulation in shoots [10, 14, 15]. In wheat, Zn deficiency increases the roots membrane permeability for Pi [33]. In this study, the dynamics of Pi transport was examined for lettuce plants grown in the presence of constant concentration of Pi (500 *μ*M) and changed concentration of Zn from 0 to 180 *μ*M using radiolabeled ^33^Pi. Our results also showed that increasing the Zn concentration had limited effect on the Pi uptake and translocation capacity of Kordaat (Figures 4(a) and 4(b)). However, increased Zn concentration reduced both Pi uptake and transfer of Pi to the shoots in Paris Island Cos (Figures 4(a) and 4(b)). This result is in line with previous studies in many plants species such as cotton [7], barley [9], and wheat [10] showing that the feedback control mechanism from the shoots was impaired thusly suppressing the uptake and translocation rate of Pi at high P concentration in the shoots under Zn deficiency. The fact of whether the low Zn content may also limit the redistribution of Pi from shoot to root needs further investigations (Figure 5). At the molecular level, genes and precise mechanisms underlying this process remain to be identified. Huang et al., 2000 have provided evidence for the involvement of the high affinity Pi transporter (PHT) in the increase of Pi uptake in barley Pi-deficient plants. These results [11, 12] showed that Zn deficiency could induce the expression of the PHT1;1 in Arabidopsis. Recently, genes that are necessary for the increase in Pi overaccumulation in response to Zn deficiency in Arabidopsis have been identified, namely, the Pi exporter* PHO1* and its homologue* PHO1;H3* [12]. In Arabidopsis,* PHO1* gene is predominantly expressed in the root vascular system and it is involved in Pi loading into root xylem.* PHO1;H3* is involved in the control of Pi accumulation in response to Zn deficiency in Arabidopsis. The fact of whether the homologue of these Arabidopsis genes (*PHO1s* and* PHTs*) in lettuce is also involved in the regulation of Pi uptake and its transfer from root to shoot under Zn deficiency is under investigation (Figure 5). This understanding on Pi metabolism is possible; thanks are due to the availability of the lettuce genome sequence, which has proved to be a great asset for the identification and characterization of the genes involved in the regulation of the Pi and Zn transport systems.

## 4. Conclusion

In conclusion, work presented is an extensive comparison of the effects of a wide set of combinatory stress conditions (+/−Zn and/or Pi) on the accumulation of Pi and Zn in two lettuce varieties, Paris Island Cos and Kordaat. This study revealed the difference between the effects of Pi and Zn supply on biomass and photosynthesis and the Pi transport in both lettuce varieties, which constitutes an opportunity towards decoding genetic basis of the Pi/Zn interaction. These observations indicate that the regulation of the Pi/Zn interaction in plants is more complex than previously thought. Further forward genetic work has to be undertaken using population obtained from the crossing between Paris Island Cos and Kordaat to identify additional key genes that regulate the Pi accumulation in shoot of lettuce varieties under Zn deficiency. This knowledge is required to fully appreciate the coregulation of Zn/Pi interaction in lettuce.

## Figures and Tables

**Table 1:** Zinc and phosphate contents in Paris Island Cos and Kordaat submitted to zinc and phosphate treatments.

**Tissues**	**Pi in shoots**		**Pi in roots**	
	Kordaat	Paris Island Cos	Kordaat	Paris Island Cos

Treatments				
0 Zn, 0 Pi	2.13 ± 0.28	2.04 ± 0.11	14.77 ± 5.62	6.18 ± 0.68
15 Zn, 0 Pi	2.61 ± 0.69	2.65 ± 1.25	8.84 ± 3.34	10.25 ± 5.53
90 Zn, 0 Pi	1.79 ± 0.21	1.80 ± 0.26	11.16 ± 1.41	9.65 ± 2.49
360 Zn, 0 Pi	2.49 ± 0.29	3.10 ± 0.97	8.14 ± 4.00	16.32 ± 2.28
1440 Zn, 0 Pi	7.24 ± 2.34	4.16 ± 1.11	32.65 ± 6.02	18.18 ± 0.45
2880 Zn, 0 Pi	15.63 ± 2.80	6.47 ± 1.74	18.39 ± 13.02	8.48 ± 4.02
0 Zn, 500 Pi	6.16 ± 0.79	11.02 ± 2.18	136.17 ± 8.19	146.78 ± 7.03
15 Zn, 500 Pi	6.11 ± 0.91	9.79 ± 0.15	144.04 ± 4.40	206.07 ± 54.35
90 Zn, 500 Pi	5.58 ± 2.49	5.81 ± 4.18	40.53 ± 10.04	48.78 ± 22.37
360 Zn, 500 Pi	8.68 ± 2.76	4.39 ± 0.81	86.48 ± 11.74	46.27 ± 7.65
1440 Zn, 500 Pi	4.73 ± 0.74	4.31 ± 0.17	90.33 ± 10.09	113.49 ± 30.62
2880 Zn, 500 Pi	18.72 ± 2.39	8.62 ± 1.57	67.59 ± 25.04	109.63 ± 23.85

	**Zn in shoots**		**Zn in roots**	

	Kordaat	Paris Island Cos	Kordaat	Paris Island Cos

0 Zn, 0 Pi	0.02 ± 0.00	0.01 ± 0.00	0.03 ± 0.02	0.02 ± 0.00
15 Zn, 0 Pi	0.03 ± 0.00	0.03 ± 0.01	0.07 ± 0.02	0.07 ± 0.01
90 Zn, 0 Pi	0.22 ± 0.04	0.11 ± 0.05	1.16 ± 0.54	0.46 ± 0.23
360 Zn, 0 Pi	0.24 ± 0.03	0.13 ± 0.07	5.74 ± 5.80	5.01 ± 1.28
1440 Zn, 0 Pi	4.60 ± 2.46	2.58 ± 0.87	200.75 ± 165.78	2642.38 ± 1942.29
2880 Zn, 0 Pi	2.99 ± 0.38	3.00 ± 0.46	512.96 ± 121.85	480.58 ± 92.58
0 Zn, 500 Pi	0.03 ± 0.04	0.01 ± 0.00	0.05 ± 0.03	0.03 ± 0.01
15 Zn, 500 Pi	0.01 ± 0.00	0.01 ± 0.00	0.08 ± 0.03	0.05 ± 0.01
90 Zn, 500 Pi	0.28 ± 0.14	0.45 ± 0.43	0.22 ± 0.07	0.15 ± 0.02
360 Zn, 500 Pi	1.30 ± 0.97	0.43 ± 0.43	0.31 ± 0.03	0.17 ± 0.02
1440 Zn, 500 Pi	0.72 ± 0.62	0.24 ± 0.12	8.32 ± 1.92	7.15 ± 246.13
2880 Zn, 500 Pi	0.38 ± 0.04	0.14 ± 0.04	263.92 ± 316.46	288.12 ± 4.39

Individual measurements were obtained from the analysis of shoots or roots collected from a pool of “*n*” plants (*n* ≥ 3). ± indicate SD.
